# Oxidative folding pathways of bovine milk β‐lactoglobulin with odd cysteine residues

**DOI:** 10.1002/2211-5463.12656

**Published:** 2019-06-20

**Authors:** Michio Iwaoka, Takumi Mitsuji, Reina Shinozaki

**Affiliations:** ^1^ Department of Chemistry School of Science Tokai University Hiratsuka‐shi Kanagawa Japan

**Keywords:** AEMTS blocking, DHS^ox^, disulfide bond rearrangement, endoproteinase Glu‐C, overoxidation

## Abstract

Bovine β‐lactoglobulin (BLG) is a major whey protein with unique structural characteristics: it possesses a free Cys thiol (SH) and two disulfide (SS) bonds and consists of a β‐barrel core surrounded by one long and several short α helices. Although SS‐intact conformational folding has been studied in depth, the oxidative folding pathways and accompanying SS formation/rearrangement are poorly understood. In this study, we used *trans*‐3,4‐dihydroxyselenolane oxide, a water‐soluble selenoxide reagent which undergoes rapid and quantitative SS formation, to determine the oxidative folding pathways of BLG variant A (BLGA) at pH 8.0 and 25 °C. This was done by characterizing two key one‐SS intermediates, a particular folding intermediate having a Cys66–Cys160 SS bond (I‐1) and a particular folding intermediate having a Cys106–Cys119 SS bond (I‐2), which have a native Cys66–Cys160 and Cys106–Cys119 SS bond, respectively. In the major folding pathway, the reduced protein (R) with abundant α helices was oxidized to I‐1, which was then transformed to I‐2 through SS rearrangement. The native protein (N) was formed by oxidation of I‐2. The redundant Cys121 thiol facilitates SS rearrangement. N is also generated from an ensemble of folding intermediates having two SS bonds (2SS) intermediates with scrambled SS bonds through SS rearrangement, but this minor pathway is deteriorative due to aggregation or overoxidation of 2SS. During oxidative folding of BLGA, α→β conformational transition occurred as previously observed in SS‐intact folding. These findings are informative not only for elucidating oxidative folding pathways of other members of the β‐lactoglobulin family, but also for understanding the roles of a redundant Cys thiol in the oxidative folding process of a protein with odd Cys residues.

**Abbreviations**
1SS: an ensemble of folding intermediates having one SS bond
2SS: an ensemble of folding intermediates having two SS bonds
AEMTS: 2‐aminoethyl methanethiosulfonate
BLGA: BLG variant A
BLGB: BLG variant B
BLG: bovine β‐lactoglobulin
DHS^ox^: *trans*‐3,4‐dihydroxyselenolane oxide
DTT^red^: dithiothreitol
eq: equivalent
GdmCl: guanidinium chloride
I‐1: a particular folding intermediate having a Cys66–Cys160 SS bond
I‐2: a particular folding intermediate having a Cys106–Cys119 SS bond
N: native BLGA
R: reduced BLGA
RP: reverse phase
SH: thiol
SS: disulfide
TFA: trifluoroacetic acid

β‐Lactoglobulin, a major whey protein present in mammalian milk, is nutritionally important but sometimes acts as a food allergen 1. Bovine β‐lactoglobulin (BLG) has been a representative target of study for decades in various fields, such as structural biology 2, 3, immunology 4, 5, and food science 6. Structurally, BLG has unique features, i.e. a rigid β‐barrel core surrounded by one long and several short α‐helices with two disulfide (SS) bonds (Cys66–Cys160 near the surface and Cys106–Cys119 in the core) and one free cysteinyl thiol (SH) of Cys121 buried in the core (Fig. 1A) 7. There are two variants of BLG. Variant A (BLGA) has Asp64 and Val118 in place of Gly64 and Ala118 of another variant, B (BLGB). These variants are in equilibrium with a hetero‐ or homo‐dimer under physiological conditions 8. The biological functions are not well understood, but it is suggested that BLG is a transporter of lipophilic molecules, such as retinol and fatty acids 9. Recently, it was found that BLG can act as a catalyst of retro‐aldol reactions *in vitro* 10.

**Figure 1:**
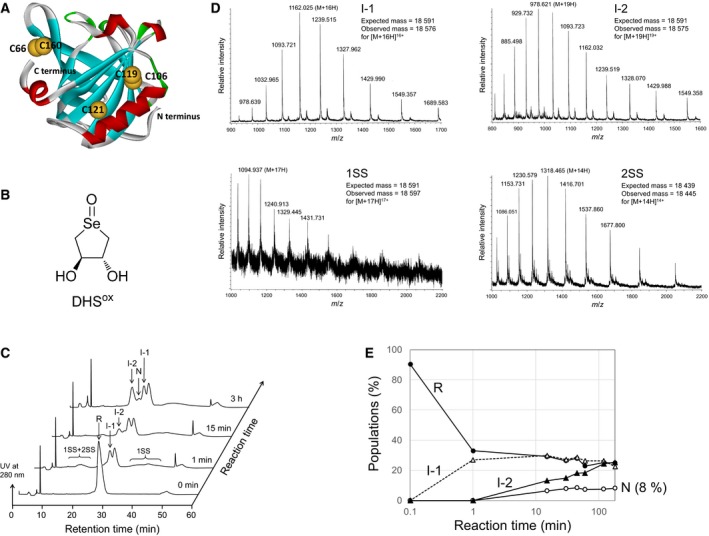
Oxidative folding of BLGA using 1 eq DHS^ox^ at pH 7.0 and 25 °C. (A) Molecular model of BLGA adopted from protein databank (code 2AKQ, chain A) 7. (B) Molecular structure of DHS^ox^. (C) RP‐HPLC chromatograms. Reaction conditions were [R]_0_ = [DHS^ox^]_0_ = 14.9 μm in 100 mm Tris pH 7.0 buffer. (D) ESI‐MS spectra for I‐1, I‐2, 1SS, and 2SS with modification of the free SH groups with AEMTS. (E) Time course of relative populations of the intermediates. Closed circle, R; open triangle, I‐1; closed triangle, I‐2; open circle, N.

The folding of BLG has attracted the interest of researchers because of its unique characteristics. By diluting a guanidinium chloride (GdmCl)‐containing denatured BLGA solution at pH 3.2 using a stopped‐flow CD instrument, Kuwajima *et al*. observed rapid accumulation of secondary structures within the dead mixing time (18 ms) 11. More detailed analysis of the SS‐intact folding of BLGA led Hamada *et al*. to conclude the occurrence of the non‐hierarchical α→β transition from the burst‐phase intermediate to the native state 12. A similar α→β transition was also observed in the SS‐intact folding of equine β‐lactoglobulin 13 as well as those of various β‐rich proteins 14, 15. Although SS‐intact folding studies have been performed elaborately 16, 17, to date the oxidative folding of BLG, accompanying SS formation and SS rearrangement, has not been reported except for one theoretical study 18. According to the energy calculation of the possible SS intermediates, it was predicted that native BLGA (N) would regenerate from the reduced state (R) via a one‐SS intermediate having a native Cys106–Cys119 SS bond. Another one‐SS intermediate having the other native SS bond, i.e. Cys66–Cys160, was suggested to be oxidized to a misfolded species by formation of a non‐native Cys106–Cys121 SS bond.

Oxidative folding of BLG would be an interesting issue on several counts. Firstly, an oxidative folding pathway of a protein with an odd number of Cys residues has not yet been reported, whereas oxidative folding pathways of proteins with even Cys residues have frequently been reported in the literature 19, 20. Secondly, the redundant (or unpaired) Cys121 residue, which can be a useful target of chemical modification of a protein 21, 22, would play roles in the oxidative folding of a protein 23, but no explicit information is currently available. Thirdly, it is of interest to see whether early formation of an α‐rich state and the transformation to a β‐rich state, as observed in the SS‐intact folding of BLGA 12, are also applicable for the SS‐coupled folding.

In this study, oxidative folding pathways of BLGA were determined by characterizing two key one‐SS intermediates, a particular folding intermediate having a Cys66–Cys160 SS bond (I‐1) and a particular folding intermediate having a Cys106–Cys119 SS bond (I‐2), at pH 8.0 and 25 °C. To enable clear observation of these intermediates, a strong and specific oxidant, *trans*‐3,4‐dihydroxyselenolane oxide (DHS^ox^) (Fig. 1B) 24, was employed. DHS^ox^ is a unique water‐soluble selenoxide reagent developed in our laboratory and has been utilized for oxidative folding of various SS‐containing proteins, such as bovine pancreatic ribonuclease A 25, 26, 27, recombinant hirudin CX‐397 28, hen egg white lysozyme 29, and bovine milk α‐lactalbumin 30, as well as native chain assembly of bovine and human insulin 31 and human relaxin‐2 31, but has never been applied to the oxidative folding of a protein with odd Cys residues. When applied to oxidative protein folding, DHS^ox^ allows rapid (< 1 min) and quantitative SS formation over a wide pH range (at least from pH 3 to 10) 24. These features are in contrast to conventional SS oxidants, such as glutathione disulfide and oxidized DTT, which make it difficult, but not impossible, to detect transiently formed key SS intermediates because by using such weak oxidants, SS formation and SS rearrangement occur competitively in the folding reaction mixture.

## Results

### Oxidative folding of BLGA using DHS^ox^

Initial attempts at oxidative folding of BLG, which contains variants A (BLGA) and B (BLGB), using DHS^ox^ revealed poor results due to severe overlapping of the peaks of the folding intermediates on the HPLC chromatograms. Therefore, the variants were separated by reverse‐phase (RP) HPLC. Purified BLGA was reduced by DTT^red^ to obtain the reduced variant (R), which was immediately reacted with DHS^ox^ under various conditions.

When the oxidative folding was carried out at pH 7.0 and 25 °C using 1 equivalent (eq) DHS^ox^, the oxidation was rapidly completed within 1 min, producing various folding intermediates, such as an ensemble of folding intermediates having one SS bond (1SS), an ensemble of folding intermediates having two SS bonds (2SS), and I‐1 (Fig. 1C), as well as a small amount of plausible overoxidized products, which eluted as a sharp peak with a washing peak. The numbers of SS bonds present in these intermediates could be easily determined by fractionation of each intermediate, subsequent blocking of the free Cys SH groups with 2‐aminoethyl methanethiosulfonate (AEMTS), and measurement of the molecular mass. Since the AEMTS‐blocking of a protein modifies the SH into SSCH_2_CH_2_NH^3+^ 32 and R possesses five SH groups along the chain, the folding intermediates having one or two SS bonds should react with three or one molecules of AEMTS, increasing the mass number by 228 and 76, respectively. Thus, 1SS and I‐1 were assigned to the intermediates having one SS bond, while 2SS was the intermediate having two SS bonds (Fig. 1D). According to the HPLC profiles, 1SS and 2SS should contain a number of species having a scrambled SS bond(s), while I‐1 would be a single species with one specific SS bond.

Although the oxidation was completed within 1 min, subsequent slow SS rearrangement was observed during prolonged incubation of the folding mixture. A time course of relative populations of the intermediates (Fig. 1E) showed slow formation of a new intermediate (I‐2) having one specific SS bond and regeneration of 8% N, suggesting that I‐2 is a key intermediate in the oxidative folding pathway of BLGA. It is also notable that broad peaks of 1SS and 2SS gradually disappeared within 3 h, while the peak of I‐1 remained explicit (Fig. 1C). This suggests that I‐1 is another important intermediate.

**Figure 2:**
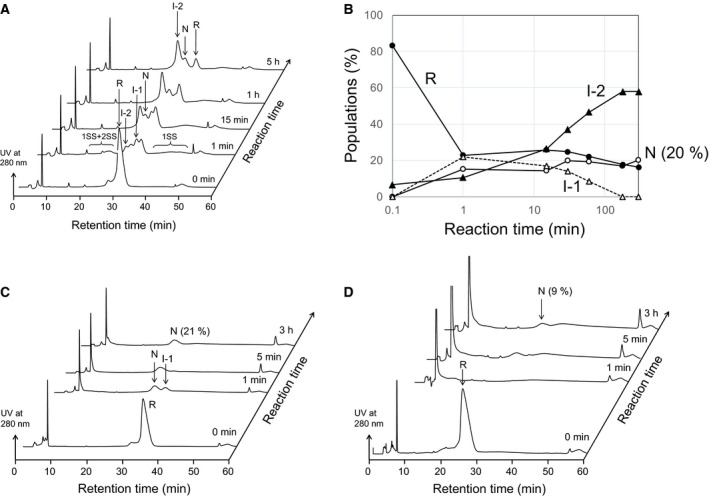
Oxidative folding of BLGA using 1–3 eq DHS^ox^ at pH 8.0 and 25 °C. (A) RP‐HPLC chromatograms obtained by using 1 eq DHS^ox^. Reaction conditions were [R]_0_ = [DHS^ox^]_0_ = 14.7 μm in 100 mm Tris pH 8.0 buffer. (B) Time course of relative populations of the intermediates under the conditions of (A). Filled circle, R; open triangle, I‐1; closed triangle, I‐2; open circle, N. (C) RP‐HPLC chromatograms obtained by using 2 eq DHS^ox^. Reaction conditions were [R]_0_ = [DHS^ox^]_0_/2 = 21.0 μm in 100 mm Tris pH 8.0 buffer. (D) RP‐HPLC chromatograms obtained by using 3 eq DHS^ox^. Reaction conditions were [R]_0_ = [DHS^ox^]_0_/3 = 21.0 μm in 100 mm Tris pH 8.0 buffer.

When the folding experiment was performed at pH 8.0, similar results but with more pronounced changes were observed due to faster SS rearrangement under a slightly basic condition (Fig. 2A,B). In this case, I‐2 was already generated within 1 min, and I‐1 almost disappeared in 5 h after incubation. According to these observations, early‐forming one‐SS intermediate I‐1 would slowly rearrange to late‐forming one‐SS intermediate I‐2. Oxidative folding of BLGB was also performed under similar conditions, but clear separation of the peaks of the folding intermediates could not be achieved (data not shown). So, folding of BLGB was not investigated further in this study.

Oxidative folding of BLGA was also carried out using 2 eq DHS^ox^ at pH 8.0 and 25 °C (Fig. 2C). Only I‐1 and N were clearly observed on the HPLC chromatograms. The presence of I‐1 after 1 min in spite of 2 eq DHS^ox^ reacting with R is indicative of a rigid structure of I‐1, which would resist the access of a DHS^ox^ molecule to the SH groups. On the other hand, absence of 2SS would be due to rapid SS rearrangement to N. The amount of N recovered after 3 h was about 21%, which was as large as that obtained by using 1 eq DHS^ox^ (20%). However, the absence of any relevant peaks other than N after 3 h on the HPLC chromatogram suggested overoxidation or aggregation of the intermediates to the polymeric species. When the oxidation was carried out using 3 eq DHS^ox^ (Fig. 2D), N was only slightly regenerated (9%), probably due to overoxidation of the folding intermediates. Oxidative folding of BLGA was also performed at different temperatures (data not shown). However, any significant temperature effects were not observed, at least between 5 and 35 °C.

### GdmCl‐induced denaturation of N

**Figure 3:**
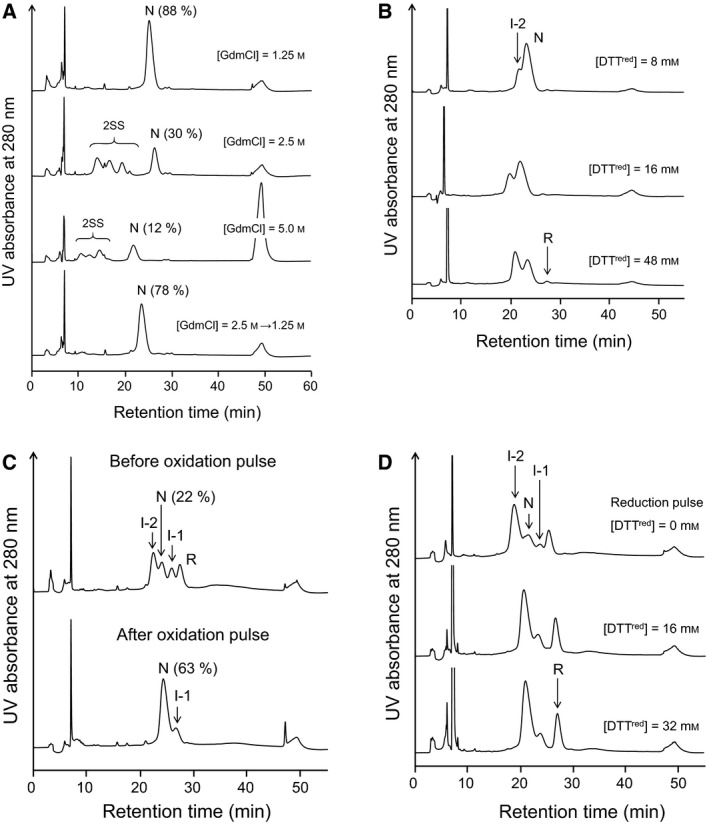
Unfolding and folding of BLGA under various reaction conditions at pH 8.0 and 25 °C. (A) RP‐HPLC chromatograms obtained by GdmCl‐induced denaturation and renaturation. Denaturation conditions were [N]_0_ = 17.2 μm in 100 mm Tris pH 8.0 buffer and denaturation time = 5 min. Renaturation condition was 5 h after 2‐fold dilution of 2.5 m GdmCl (bottom chromatogram). (B) RP‐HPLC chromatograms obtained by reductive unfolding using DTT^red^. Reduction conditions were [N]_0_ = 15.6 μm in 100 mm Tris pH 8.0 buffer and reaction time = 3 min. (C) RP‐HPLC chromatograms obtained by oxidation pulse experiment. Oxidative folding conditions were [R]_0_ = [DHS^ox^] = 15.8 μm in 100 mm Tris pH 8.0 buffer and reaction time = 30 min. Oxidation pulse conditions were 1 eq DHS^ox^ and reaction time = 3 min. (D) RP‐HPLC chromatograms obtained by reduction pulse experiment. Oxidative folding conditions were [R]_0_ = [DHS^ox^] = 10.0 μm in 100 mm Tris pH 8.0 buffer and reaction time = 3 h. Reduction pulse conditions were [DTT^red^] = 0–32 mm and reaction time = 3 min.

BLGA in the native state (N) has one free SH (Cys121), which is buried near the β‐barrel core but would be capable upon denaturation of reacting intramolecularly with the SS bonds to reshuffle them 33. Indeed, when N was incubated at pH 8.0 and 25 °C in the presence of 2.5 m GdmCl, formation of SS‐reshuffled intermediates (2SS) was observed, leaving 30% of N (Fig. 3A). Interestingly, the 2SS species disappeared to regenerate N when the solution was diluted with the same volume of the buffer solution, suggesting that the conversion from 2SS to N can be a possible pathway to N in the oxidative folding of BLGA. In this experiment, the recovery yield of N was 78% based on the integrated peak area. On the other hand, when N was incubated in the presence of 5.0 m GdmCl, a large peak was observed with a washing peak on the HPLC chromatogram, suggesting aggregation of 2SS. Since this peak remained after diluting the solution, 2SS would have a significant propensity to produce polymeric species, which should not be preferable for efficient regeneration of N from R.

### Reduction of N

When N was reduced at pH 8.0 and 25 °C with excess DTT^red^ (8–48 mm), formation of I‐2 was observed (Fig. 3B), confirming that I‐2 possesses one native SS bond and is a direct precursor of N on the oxidative folding pathways of BLGA. When the concentration of DTT^red^ was increased up to 48 mm, formation of a small amount of R was observed, but I‐1 was never observed in these experiments. The results suggested that the two native SS bonds of N are selectively reduced by DTT^red^ step‐by‐step to generate R.

### Oxidation and reduction pulse experiments 27

To obtain more information on the intermediates generated during the oxidative folding of BLGA, an oxidation pulse (1 eq DHS^ox^, 3 min) or a reduction pulse (16 or 32 mm DTT^red^, 3 min) was given to the reaction mixture of R and 1 eq DHS^ox^ at pH 8.0 and 25 °C after a certain period of incubation. When the oxidation pulse was applied to the reaction mixture after 30 min, the peaks of R and I‐2 disappeared, while the peak of N increased in the intensity (Fig. 3C), suggesting that R and I‐2 are oxidized to I‐1 (or 1SS) and N, respectively. The yield of N was estimated to be up to 63% after the oxidation pulse. The peak of I‐1 did not show a significant change in intensity. This would be due to compensation of the formation of I‐1 by oxidation of R with a loss of I‐1 by oxidation itself.

On the other hand, when the reduction pulse was applied to the reaction mixture after 3 h, the peaks of N and I‐1 decreased, while the peaks of I‐2 and R increased in intensity (Fig. 3D), suggesting that N and I‐1 are reduced to I‐2 and R, respectively.

### Structural characterization of I‐1 and I‐2

**Figure 4:**
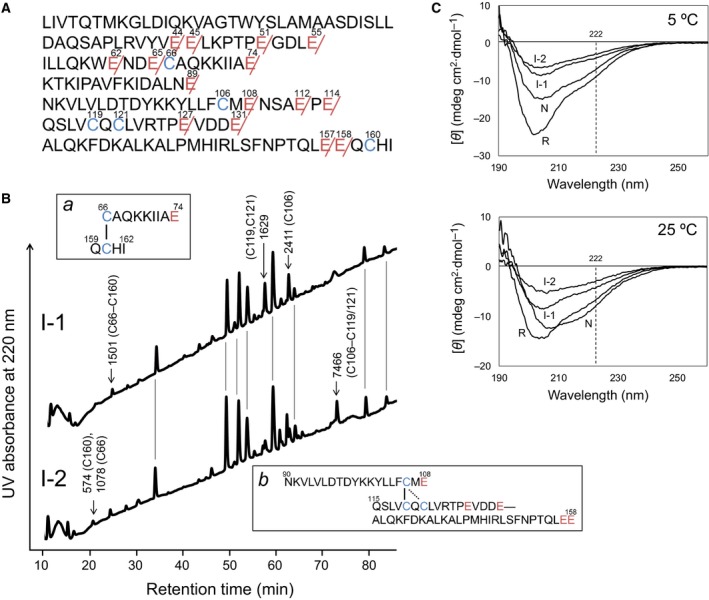
Characterization of I‐1 and I‐2. (A) The amino acid sequence of BLGA with the positions of the peptide bonds, which should be hydrolyzed by Glu‐C, indicated. (B) RP‐HPLC chromatograms obtained by Glu‐C digestion of I‐1 and I‐2. Molecular masses determined by LC‐MS are indicated for representative fragments having cysteine residues along with amino acid sequences for the fragment of I‐1 (M^+^_obs_ = 1501) and that for the fragment of I‐2 (M^+^_obs_ = 7466) are shown in insets *a* and *b*, respectively. (C) CD spectra for AEMTS‐blocked R, I‐1, I‐2, and N measured in 10 mm Tris pH 8.0 buffer at 5 or 25 °C. Concentration of BLGA was 6.9 μm.

The positions of the SS bond of I‐1 and I‐2 could be assigned to Cys66–Cys160 and Cys106–Cys119, respectively, as follows. The AEMTS‐blocked intermediates, as well as N and R, were digested with endoproteinase Glu‐C, which site‐specifically hydrolyzes Glu–Xaa peptide bonds 34. In the case of BLGA, which has 15 Glu residues, the Glu‐C digestion should produce a number of peptide fragments (Fig. 4A), the amino acid sequence of which can be assigned based on the mass numbers determined by LC‐MS analysis. To do this, AEMTS blocking is helpful because AEMTS modification of a cysteinyl SH group shifts the mass number by +76, which allows easy assignment whether the peptide fragments have SH or SS groups.

When I‐1 and I‐2 were digested by Glu‐C, more than 20 peptide fragments were obtained (Fig. 4B). The HPLC chromatograms showed they had almost identical profiles to one other, which were also similar to those obtained from the Glu‐C digestion of R and N (Fig. S1), with a number of common fragments. Taking a closer look, however, one could notice the presence or absence of a few fragments in each chromatogram. Structural assignments of the representative peptide fragments are summarized in Tables S1–S4.

Among the fragments obtained from I‐1, the peptides having Cys66–Cys160 (M^+^_obs_ = 1501) (inset *a* of Fig. 4B), Cys119, Cys121 (M^+^_obs_ = 1629), and Cys106 (M^+^_obs_ = 2411) were found. Thus, I‐1 could be assigned to a one‐SS intermediate having a native Cys66–Cys160 SS bond. Similarly, the peptides having Cys66 (M^+^_obs_ = 1078), Cys160 (M^+^_obs_ = 574) and Cys106–Cys119/Cys121 (M^+^_obs_ = 7468) (inset *b* of Fig. 4B) were found in the fragments obtained from I‐2. Thus, I‐2 was assigned to a one‐SS intermediate having either a native Cys106–Cys119 or a non‐native Cys106–Cys121 SS bond. The exact position of the SS bond of I‐2 could not be determined according to the Glu‐C digestion analysis. Nevertheless, the native Cys106–Cys119 SS bond is most likely because I‐2 is a direct precursor of N as evidenced by the reduction experiment of N using DTT^red^ (Fig. 3B) and the oxidation pulse experiment (Fig. 3C).

The CD spectra of AEMTS‐blocked I‐1 and I‐2 measured at pH 8.0 and 5 and 25 °C are shown in Fig. 4C along with those of AEMTS‐blocked R and N. It is of note that R had a comparable number of or more α helices than N, even though R was modified with five molecules of AEMTS, based on the significant negative CD signal at 222 nm. In contrast, I‐1 and I‐2 did not have helical structure. The contents of α helices at 5 °C were roughly estimated, by utilizing the mean residue molecular ellipticity at 208 nm ([θ]_208_) 35, to be 47, 10, 5, and 29% for R, I‐1, I‐2, and N, respectively, suggesting that during the oxidative folding of BLGA, α helices vanished at the beginning and then reformed in the last oxidation step. Similar trends were observed in the CD spectra at 25 °C, but the α contents were monotonously decreased to 26, 10, 1, and 24% for R, I‐1, I‐2, and N, respectively. It should be noted that the α content estimated for N (24%) is consistent with that calculated for the native structure shown in Fig. 1A (23%).

## Discussion

### Oxidative folding pathways of BLGA

**Figure 5:**
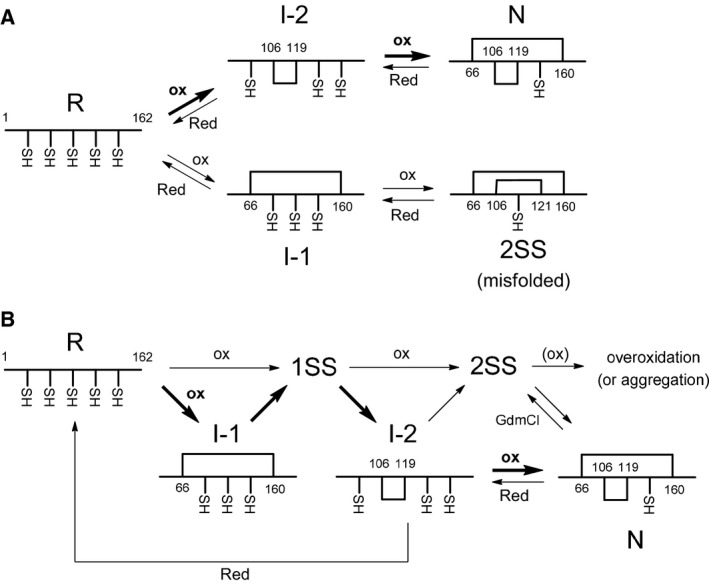
Oxidative folding pathways of BLGA. (A) Predicted by theoretical calculation 18. (B) Determined in this study. Major folding pathways are shown with thick arrows.

Although the conformational folding of BLGA with the native SS bonds intact has been extensively elucidated under acidic conditions 11, 12, 16, 17, an oxidative folding study has not been reported to date. In the meantime, a theoretical study predicted the presence of two specific intermediates as shown in Fig. 5A 18. They are both one‐SS intermediates with one native SS bond, which correspond to I‐1 and I‐2, respectively, observed in this study. I‐2 with an inner Cys106–Cys119 SS bond was predicted to be an on‐pathway intermediate, which would be oxidized to N. I‐1 with an outer Cys66–Cys160 SS bond was predicted to be an off‐pathway intermediate, which would be oxidized to misfolded 2SS species. However, these predictions had not been supported by any experimental evidence.

In this study, by exploiting advantageous features of DHS^ox^ as an oxidative folding reagent 24, we succeeded in characterizing the two key SS intermediates on the folding pathways of BLGA for the first time. The oxidative folding pathways of BLGA determined in this study are illustrated in Fig. 5B. Starting from R, the oxidation generates a key one‐SS intermediate (I‐1) as well as an ensemble of 1SS having a scrambled SS bond. Then, I‐1 is transformed to another key one‐SS intermediate (I‐2) through SS rearrangement. Since I‐1 and I‐2 have an SS bond in a different position, the SS rearrangement should take place via 1SS. Finally, I‐2 is oxidized to N. This pathway would be a major route from R to N. There is another pathway, which goes through an ensemble of the 2SS intermediates, but this route would not be preferable because 2SS has a propensity to aggregate or overoxidize irreversibly to polymeric species as observed in the oxidative folding using excess amounts of DHS^ox^ (Fig. 2D) and GdmCl‐induced denaturation of N (Fig. 3A). In the reductive unfolding of BLGA, N should be reduced to R through I‐2, which has an outer native SS bond located near the surface having been reduced. It should be noted that dimer formation of N may interfere with these folding pathways. However, the dimer should be predominant at a concentration higher than 45 μm 8. Therefore, the equilibrium between the monomer and dimer would prefer the monomeric state in the folding experiments performed in this study.

The folding pathways of BLGA revealed in this study have similar features to those theoretically predicted 18 in that I‐1 and I‐2 are involved as key intermediates and N is formed by oxidation of I‐2. However, in the previous prediction I‐1 was an off‐pathway intermediate, formation of which is deteriorative for correct folding of BLGA. On the other hand, I‐1 is located on‐pathway in the revised pathways. Formation of I‐1 would probably prevent the folding intermediates of BLGA from undesired aggregation, thus guiding the unfolded peptide chain to the native fold effectively.

The major folding pathway of BLGA (Fig. 5B) shows an unusual feature that the outer Cys66–Cys160 SS bond forms earlier in spite of a larger chain entropic cost than the inner Cys106–Cys119 SS bond formation. This can be explained by considering the non‐native structure present in the unfolded R state as discussed below. Moreover, this native SS bond between Cys66 and Cys160 is cleaved in the next stage of the folding. Breaking of an early‐forming native SS bond in the subsequent folding stage has sometimes been observed in the oxidative folding of other SS‐containing proteins. For example, on the major oxidative folding pathways of bovine pancreatic trypsin inhibitor, which has six Cys residues, the solvent‐exposed native Cys14–Cys38 SS bond forms first but it should be cleaved before reaching the native state 36.

### Comparison to the SS‐intact folding

According to CD spectral analysis (Fig. 4C), it is clear that R contains a significant number of α helices. This is consonant with the burst‐phase intermediate observed in the SS‐intact conformational folding of BLGA, where the α‐rich burst‐phase intermediate promptly forms and gradually transforms to the β‐rich native state 12. A similar non‐hierarchical folding scenario can be delineated in the major oxidative folding pathway revealed in this study.

The α‐helix rich R would have a collapsed structure, which may prevent formation of an entropically favorable inner Cys106–Cys119 SS bond. Instead, it would bring the Cys66 near the C‐terminal Cys160 residue so that the SS bond is easily formed between these residues to generate I‐1. Once this SS bond is formed, the peptide chain would gain thermodynamic stability making a compact structure, which would prevent further oxidation of the remaining Cys SHs to a substantial extent. We propose that there are some α helices still in I‐1 but they could not be detected for AEMTS‐blocked I‐1 in the CD spectrum measured at pH 8.0. Indeed, the CD spectrum for acid‐quenched I‐1 measured in 20 mm HCl at 5 °C suggested the presence of about 30% α helices on average (data not shown), which is comparable to that in the burst‐phase intermediate of BLGA in the SS‐intact folding (38%) 12. Subsequently, SS reshuffling would take place in I‐1 to seek another potential energy minimum structure, which should be assigned to I‐2. During this process, α→β conformational transition would proceed, accompanied by formation of a β‐barrel core. Although the presence of the β‐barrel structure in I‐2 is not evidenced in this study, the formation is likely because the Cys106–Cys119 SS bond locks two strands of the β‐barrel core in the native state (Fig. 1A). Finally, I‐2 is oxidized to N, reforming the Cys66–Cys160 SS bond as well as α helices.

The above scenario of the oxidative folding of BLGA is consonant to the SS‐intact folding 12. Thus, the α→β conformational transition would be intrinsic to the nature of the amino acid sequence of BLG, suggesting a similar conformational transition takes place during the folding in cells.

### Roles of a redundant Cys121 residue

A free cysteinyl SH present in a folded protein frequently plays important biological roles as an active center of cysteine proteases and a resolving SH group to support the catalytic cycle of oxidoreductase enzymes 37, 38. It is also utilized frequently as a target of chemical modification of a protein with various functional moieties 21, 22. However, the possible roles in protein folding are not well known. In the case of BLG, the redundant Cys121 residue is buried in the core, so it is unlikely that this residue has important biological functions. Instead, possible roles in stabilization of the folded structure 39 and oxidative folding 23 have been suggested. In the major folding pathway (Fig. 5B), the transition from I‐1 to I‐2 is a key step. This SS rearrangement step can be achieved with the aid of intramolecular free SH groups. If Cys121 is not present, the transition would be significantly distorted, thereby reducing efficiency of the folding to N. This would be in agreement with the previous observation by Invernizzi *et al*. that the C121S mutant of BLG is more prone to aggregation than is the wild‐type protein upon intracellular expression 23, indicating an important role of redundant Cys121 in the oxidative folding of BLG. The presence of Cys121 would also support the SS rearrangement process from 2SS to N. However, Cys121 can be deteriorative simultaneously due to overoxidation of 2SS 33. These roles of redundant Cys121 in the oxidative folding of BLGA, whether positive or negative, are not clearly evidenced in this study. The oxidative folding of BLGA with mutation of the Cys121 residue to other amino acids 33 or the modification with a SH‐blocking reagent 40 will provide useful information about this point in future study.

The oxidative folding pathways of BLGA (Fig. 5B) revealed in this study share common features with those predicted previously (Fig. 5A) 18. However, there are distinct differences between them. In our pathways, R with abundant α helices is oxidized to I‐1, which is then transformed to I‐2 through SS rearrangement. Both I‐1 and I‐2 have a native SS bond but the positions are different. Finally, I‐2 is oxidized to N. During this major folding process, α→β conformational transition should occur as previously observed in the SS‐intact folding of BLGA 12. The redundant SH of Cys121 may play a role to facilitate SS rearrangement during the transformation from I‐1 to I‐2. In addition to this major pathway, there would be a bypass route to N, which goes through 2SS species. This route, however, should be deteriorative simultaneously because of aggregation or overoxidation of 2SS. These findings will be informative not only for elucidating oxidative folding pathways of other members of β‐lactoglobulin family but also for understanding the roles of a redundant cysteinyl SH in the oxidative folding process of a protein with odd Cys residues.

## Materials and methods

### General

β‐Lactoglobulin from bovine milk, which contains variants A and B, was purchased from Sigma‐Aldrich (Tokyo, Japan). BLGA and BLGB were purified by HPLC through a semi‐preparative RP column (Mightysil RP‐18, 10 × 250 mm; Kanto Chemical, Tokyo, Japan) applying a gradient of acetonitrile (from 41% to 46% in 17 min at a flow rate of 2 mL·min^−1^ using 0.1% trifluoroacetic acid (TFA) in H_2_O and 0.1% TFA in acetonitrile as eluents). The fractionated variants were lyophilized and stored at −30 °C. DHS^ox^ 41 and AEMTS 42 were synthesized according to methods in the literature. Endoproteinase Glu‐C from *Staphylococcus aureus* V8 was obtained from Sigma‐Aldrich. All other reagents were commercially available from local suppliers and used without further purification. Buffer solutions were prepared according to standard protocols and purged thoroughly with nitrogen gas before use.

### Preparation of reduced BLGA (R)

To a solution of BLGA (ca. 8 mg) in 0.6 mL of 100 mm Tris–HCl/1 mm EDTA buffer solution at pH 8.5 containing 5 m GdmCl was added an excess amount of DTT^red^ (42 mg). The mixture was incubated at room temperature for 2 h. The resulting solution was diluted 2‐fold with 0.6 mL of a 100 mm Tris–HCl/1 mm EDTA buffer solution at pH 8.0 (or 7.0) and passed through a column packed with Sephadex G25 resin, which was equilibrated with the same Tris buffer solution. The fraction containing R was collected, and the concentration was determined by UV measurement at 280 nm using the molar extinction coefficient of 18 700 m ^−1 ^cm^−1^, which had been determined beforehand by amino acid analysis. The R solution was immediately used for the oxidative folding experiments as described below. A similar procedure was applied for preparation of reduced BLGB.

### Oxidative folding using DHS^ox^

A DHS^ox^ solution with a concentration of 1‐, 2‐, or 3‐fold with respect to R was prepared using a 100 mm Tris–HCl/1 mm EDTA buffer solution at pH 8.0 (or 7.0) and maintained at 25.0 ± 0.1 °C in a dry thermo bath. The R solution (100 μL each) as prepared above was put into eight to ten 1.5 mL microtubes maintained at 25.0 ± 0.1 °C in a dry thermo bath. To each microtube was added 100 μL of the DHS^ox^ solution. After incubation for 1 min to 24 h at the same temperature, 15 μL of 1 m HCl was added to the microtube to quench the reaction. After 10 min, 800 μL of 0.1% TFA in H_2_O was added to the mixture. The sample solutions were stored at −30 °C till HPLC analysis.

### GdmCl‐induced denaturation of native BLGA

BLGA (ca. 1 mg) was dissolved in 2.0 mL of a 100 mm Tris–HCl/1 mm EDTA buffer solution at pH 8.0. The solution (100 μL each) was put into several 1.5 mL microtubes, maintained at 25.0 ± 0.1 °C, and to it was added 100 μL of 100 mm Tris–HCl/1 mm EDTA buffer solution at pH 8.0 containing 2.5, 5.0, or 10.0 m GdmCl. After incubation for 5 min, 15 μL of 1 m HCl and then 800 μL of 0.1% TFA in H_2_O were added to the mixture. Alternatively, the mixture was diluted 2‐fold with a 100 mm Tris–HCl/1 mm EDTA buffer solution at pH 8.0 without GdmCl and further incubated for 5 h before quenching with 1 m HCl. The sample solutions were stored at −30 °C.

### Oxidation and reduction pulse experiments

Oxidative folding of BLGA was carried out at pH 8.0 and 25 °C using 1 eq DHS^ox^ as described above. After incubation for 30 min, 100 μL of the DHS^ox^ solution (1 eq) was added to the reaction mixture (200 μL) as an oxidation pulse. Alternatively, 200 μL of 32 or 64 mm DTT^red^ in a 100 mm Tris–HCl/1 mm EDTA buffer solution at pH 8.0 was added as a reduction pulse after incubation for 3 h. The resulting mixtures were further incubated for 3 min, 15 μL of 1 m HCl and then 800 μL of 0.1% TFA in H_2_O were added to the mixture. The sample solutions were stored at −30 °C.

### Reductive unfolding of N using DTT^red^

BLGA (ca. 1 mg) was dissolved in 2.0 mL of a 100 mm Tris–HCl/1 mm EDTA buffer solution at pH 8.0. To this solution (100 μL) in a 1.5 mL microtube maintained at 25.0 ± 0.1 °C was added 100 μL of 16, 32, or 96 mm DTT^red^ in a 100 mm Tris–HCl/1 mm EDTA buffer solution at pH 8.0. After incubation for 3 min, 15 μL of 1 m HCl and then 800 μL of 0.1% TFA in H_2_O were added to the mixture. The sample solutions were stored at −30 °C.

### HPLC analysis

The acid‐quenched sample solutions obtained from the above experiments were thawed and analyzed with a Shimadzu LC‐10Ai HPLC system equipped with a 1 mL sample solution loop and an analytical RP column (TSKgel ODS‐100V 4.6 × 150 mm; Tosoh, Tokyo, Japan), which was equilibrated at 25 °C with 59 : 41 (v/v) mixture of 0.1% TFA in H_2_O (eluent A) and 0.1% TFA in acetonitrile (eluent B) at a flow rate of 0.5 mL·min^−1^. After injection of the sample solution (1 mL), a gradient of acetonitrile (the ratio of eluent B linearly increased from 41% to 45% in 0–40 min and from 45% to 100% in 40–42 min) was applied. The folding intermediates were detected by UV (SPD‐M10A vp; Shimadzu, Kyoto, Japan) at 280 nm. The recorded signals were integrated and analyzed by using lc solution software (Shimadzu).

### Blocking of the folding intermediates with AEMTS

AEMTS‐blocked folding intermediates for ESI‐MS analysis, Glu‐C digestion, and CD measurement were prepared as follows. Oxidative folding of BLGA was repeatedly carried out using 1 eq DHS^ox^ at pH 8.0 and 5 °C. After incubation for 1 min (for isolation of I‐1) or 3 h (for isolation of I‐2), 15 μL of 1 m HCl and then 800 μL of 0.1% TFA in H_2_O were added to the reaction mixture, which was then stored at −30 °C. The acid‐quenched sample solutions were thawed and injected onto the HPLC system equipped with a 1 mL sample solution loop and an analytical RP column (Zorbax 300SBC‐18 4.6 × 150 mm; Agilent, Santa Clara, CA, USA) equilibrated at 25 °C with a 60 : 40 (v/v) mixture of 0.1% TFA in H_2_O (eluent A) and 0.1% TFA in acetonitrile (eluent B) at a flow rate of 0.5 mL·min^−1^. A gradient of acetonitrile (the ratio of eluent B linearly increased from 40% to 48% in 0–40 min and from 48% to 100% in 40–42 min) was applied. The folding intermediates were fractionated by monitoring UV absorbance at 280 nm. The collected solutions were separately lyophilized. The purified acid‐quenched intermediates were dissolved in 100 μL of an AEMTS solution (1.3 mg·mL^−1^) in 100 mm acetate buffer at pH 5.0 containing 5 m GdmCl and 1 mm EDTA at 25 °C. After incubation for 1 h, 15 μL of 1 m HCl and then 800 μL of 0.1% TFA in H_2_O were added to the mixture. The AEMTS‐blocked intermediates were desalted and purified by HPLC under the same conditions as above. The collected fractions were lyophilized and stored at −30 °C. Quantities of the folding intermediates were determined by amino acid analysis.

Samples of AEMTS‐blocked N and R were prepared by mixing the same volumes (100 μL each) of a N (or R) solution (ca. 1 mg·mL^−1^) in 100 mm Tris–HCl/1 mm EDTA buffer at pH 8.0 and an AEMTS solution (10 mg·mL^−1^) in the same buffer at 25 °C. After incubation for 30 min, 15 μL of 1 m HCl and then 800 μL of 0.1% TFA in H_2_O were added to the reaction mixture. The AEMTS‐blocked N and R were purified by HPLC under the same conditions as described above. The collected fractions were lyophilized and stored at −30 °C. The quantities were determined by amino acid analysis.

### LC‐MS analysis

The molecular masses for AEMTS‐blocked N, R, and folding intermediates (I‐1, I‐2, 1SS, and 2SS) were measured on a Jeol JMS‐T100LP mass spectrometer (Jeol, Akishima, Japan), operated in the ESI(+) mode, connecting to an Agilent 1200 series HPLC system. Each sample, which had a different molecular mass depending on the number of free SH groups having been modified with AEMTS, was dissolved in a 1 : 1 mixture of 0.1% formic acid in H_2_O and 0.1% formic acid in acetonitrile and injected onto the LC‐MS system. Angiotensin I was employed as a standard.

### Glu‐C digestion

The positions of SS linkages in folding intermediates I‐1 and I‐2 were determined by enzymatic digestion using endoproteinase Glu‐C. The AEMTS‐blocked intermediates (2–4 nmol), as well as AEMTS‐blocked N and R, were separately dissolved in 100 μL of 50 mm NH_4_HCO_3_ in H_2_O. To the solution was added 1 weight % Glu‐C dissolved in H_2_O. After incubation at 37 °C for 18 h, the digestion was quenched by addition of 12 μL of 5% TFA in H_2_O. The digested sample solutions were injected onto the LC‐MS system equipped with a 20 μL sample solution loop and an analytical RP column (Zorbax 300SBC‐18 4.6 × 150 mm; Agilent) equilibrated at 25 °C with 93 : 7 (v/v) mixture of 0.1% formic acid in H_2_O (eluent A) and 0.1% formic acid in acetonitrile (eluent B) at a flow rate of 0.2 mL·min^−1^. A gradient of acetonitrile (the ratio of eluent B linearly increased from 7% to 40% in 0–72 min and from 40% to 100% in 72–74 min) was applied. The fractionated peptide fragments were monitored by UV at 220 nm. The sample was ionized with the ESI(+) mode, and the ionized fragments were monitored in a range of *m*/*z* 100–5000.

### CD measurement

The AEMTS‐blocked I‐1, I‐2, N, and R were dissolved in a 10 mm Tris–HCl/1 mm EDTA buffer solution at pH 8.0 to make solutions of 6.9 μm concentration. The solution was poured into a quartz cell with a light path length of 1 mm, which was set in a cell holder of a Jasco J‐820 CD spectrometer (Jasco, Hachioji, Japan). The temperature of the cell was thermostated at 5.0 or 25.0 ± 0.1 °C with a circular water bath. The CD spectrum was measured with a wavelength from 190 to 260 nm, a scan speed of 50 nm·min^−1^, a response of 1 s, and an accumulation number of 16 times.

## Conflict of interest

The authors declare no conflict of interest.

## Author contributions

MI and RS designed this research. TM carried out most of the experiments with the support of RS. All authors read and approved the final manuscript.

## Supporting information


**Fig. S1.** RP‐HPLC chromatograms obtained by Glu‐C digestion of R and N along with those of I‐1 and I‐2.
**Table S1.** Structure assignments of the fragments obtained by Glu‐C digestion of I‐1.
**Table S2.** Structure assignments of the fragments obtained by Glu‐C digestion of I‐2.
**Table S3.** Structure assignments of the fragments obtained by Glu‐C digestion of R.
**Table S4.** Structure assignments of the fragments obtained by Glu‐C digestion of N.
