# Supplementary material for: Oxidative folding pathways of bovine milk β‐lactoglobulin with odd cysteine residues
Source: FEBS Open Bio. 2019 Jun 20;9(8):1379–91. doi: 10.1002/2211-5463.12656 (PMC6668375; doi:10.1002/2211-5463.12656)
Supplement: Supplementary file 1 — Fig. S1. RP‐HPLC chromatograms obtained by Glu‐C digestion of R and N along with those of I‐1 and I‐2. Table S1. Structure assignments of the fragments obtained by Glu‐C digestion of I‐1. Table S2. Structure assignments of the fragments obtained by Glu‐C digestion of I‐2. Table S3. Structure assignments of the fragments obtained by Glu‐C digestion of R. Table S4. Structure assignments of the fragments obtained by Glu‐C digestion of N. [file FEB4-9-1379-s001.pdf]

Supporting Information

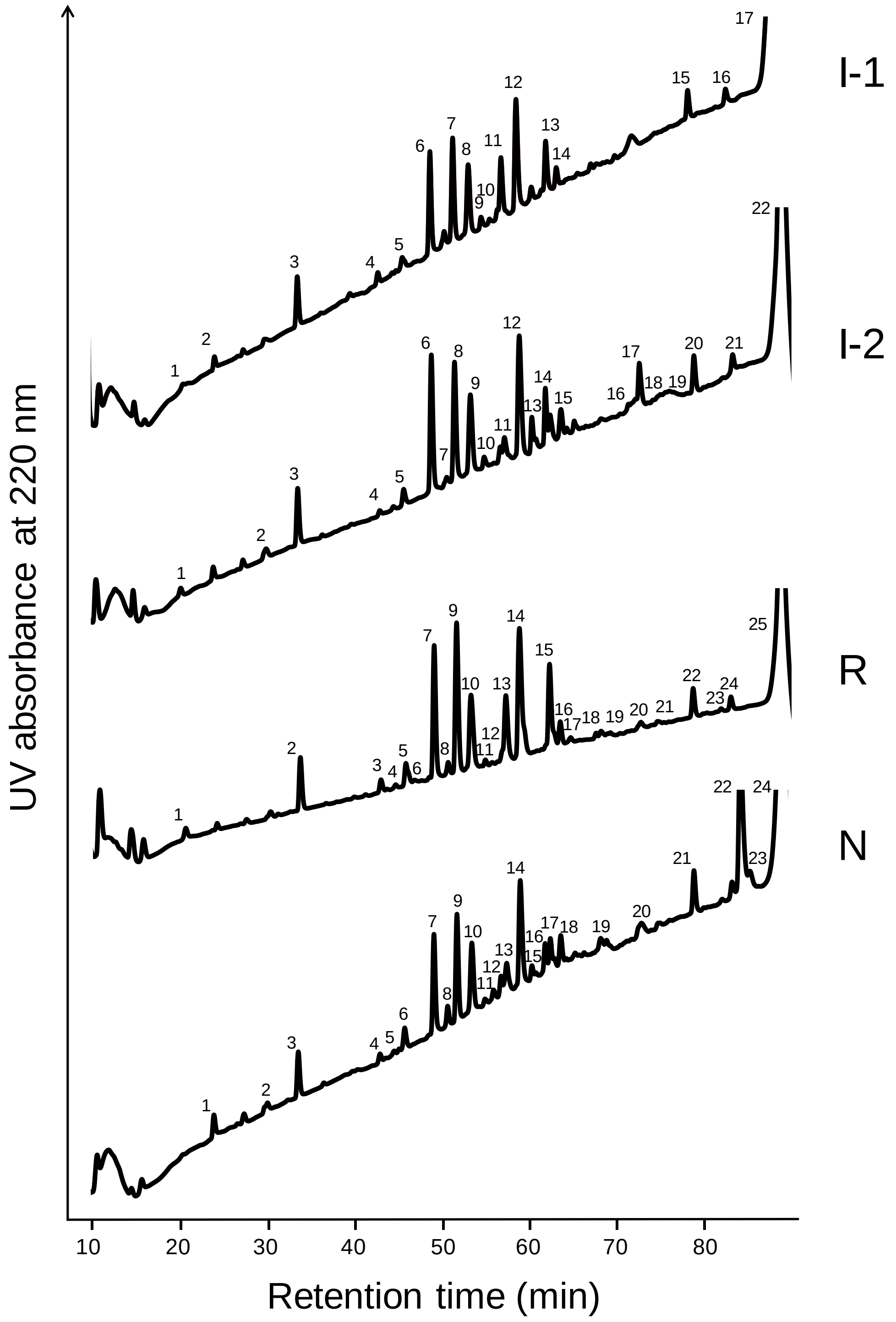

Fig. S1. RP-HPLC chromatograms obtained by Glu-C digestion of R and N along with those of I-1 and I-2.

Table S1. Structure assignments of the fragments obtained by Glu-C digestion of I-1.

Peak No.	HPLC Reaction time (min)	Observed MS	Estimated MS	Fragment	Expected MS	Cys or SS
1	9.984	540.38 (2+)<br>**646.37 (1+)**	1078.78<br>**645.37**	66-74?<br>**109-114**	1079.36<br>**645.62**	Cys66?
2	10.568	540.45 (2+)<br>**751.53 (2+)**	1078.9<br>**1501.07**	66-74?<br>**66-74=159-162**	1079.36<br>**1500.79**	Cys66?<br>**Cys66-Cys160**
3	34.322	**549.89 (2+)**<br>857.53 (1+)	**1097.77**<br>856.53	**46-55**	**1098.21**	
4	43.063	455.37 (1+)<br>554.49 (1+)<br>800.66 (1+)	454.37<br>553.49<br>799.66			
5	45.886	681.49 (2+)	1360.98			
6	49.040	**644.46 (2+)**	**1286.92**	**56-65**	**1287.42**	
7	51.628	**465.41 (2+)**<br>**929.79 (1+)**<br>590.48 (1+)<br>703.48 (1+)<br>816.65 (1+)	**928.80**<br>589.48<br>702.48<br>815.62	**56-62**	**929.12**	
8	52.932	**563.47 (3+)**<br>**844.14 (2+)**<br>657.57 (2+)<br>713.67 (2+)<br>771.21 (2+)	**1686.84**<br>1313.14<br>1425.34<br>1540.42	**75-89**	**1686.99**	
9	55.178	798.29 (7+)<br>931.10 (6+)	5580.82			
10	56.010	668.77 (5+)<br>835.48 (4+)	3338.39			
11	56.748	**544.07 (3+)**<br>**815.57 (2+)**<br>1123.64 (2+)	**1629.18**<br>2245.28	**115-127**	**1628.02**	**Cys119, Cys121**
12	58.013	707.77 (2+)<br>1415.17 (1+)	1414.07			
13	61.294	**603.66 (4+)**<br>**804.62 (3+)**	**2410.75**	**90-108**	**2411.92**	**Cys106**
14	62.383	603.66 (2+)<br>952.61 (2+)	1205.32<br>1903.22			
15	80.634	**1094.68 (17+)**<br>**1163.07 (16+)**	**18595.20**	**1-162**	**18597.17**	**Cys66-Cys160,**<br>**Cys106, Cys119, Cys121?**
16	85.700	**1225.55 (4+)**<br>**1633.54 (3+)**	**4897.90**	**1-45**	**4897.63**	

Table S2. Structure assignments of the fragments obtained by Glu-C digestion of I-2.

Peak No.	HPLC Reaction time (min)	Observed MS	Estimated MS	Fragment	Expected MS	Cys or SS
1	10.675	539.89 (2+)<br>1079.81 (1+)<br>575.35 (1+)<br>684.48 (1+)	1078.30<br>574.32<br>683.48	66–74<br>159–162<br>46–51	1079.36<br>575.73<br>683.79	Cys66<br>Cys160
2	31.637	549.87 (2+)<br>659.48 (6+)<br>790.49 (5+)<br>857.70 (1+)	1097.74<br>3950.88<br>856.70	46–55	1098.21	
3	34.369	549.88 (2+)<br>659.50 (1+)<br>857.53 (1+)	1097.76<br>658.50<br>856.53	46–55	1098.21	
4	43.214	455.37 (1+)<br>554.49 (1+)<br>800.66 (1+)	454.37<br>553.49<br>799.66			
5	45.808	681.52 (2+)	1361.04			
6	49.059	644.46 (2+)	1286.92	56–65	1287.42	
7	50.428	560.45 (1+)<br>737.52 (2+)	559.45<br>1473.04			
8	51.665	465.39 (2+)<br>929.79 (1+)<br>590.38 (1+)<br>703.48 (1+)<br>816.65 (1+)	928.79<br>589.39<br>702.48<br>815.65	56–62	929.12	
9	53.014	563.16 (3+)<br>844.12 (2+)<br>657.57 (2+)<br>713.59 (2+)	1686.36<br>1313.14<br>1425.18	75–89	1686.99	
10	56.073	668.61 (5+)<br>835.36 (4+)	3337.76			
11	56.742	1123.66 (2+)	2245.32			
12	57.970	707.77 (2+)<br>1415.06 (1+)	1414.06			
13	59.120	707.81 (4+)<br>943.80 (3+)	2827.83			
14	61.339	603.66 (4+)<br>804.63 (3+)<br>932.49 (5+)<br>1165.65 (4+)	2410.76<br>4658.60	90–108?	2411.92	Cys106?
15	62.519	952.60 (2+)	1903.20			
16	63.716	774.47 (6+)<br>929.41 (5+)<br>1161.19 (4+)	4641.21			
17	69.517	830.59 (9+)<br>934.54 (8+)<br>1067.92 (7+)<br>1245.54 (6+)	7467.53	90–108=115–158	7465.72	Cys106–Cys119,<br>Cys121
18	70.607	898.65 (9+)<br>1010.45 (8+)<br>1154.72 (7+)<br>1347.17 (6+)	8075.60	90–158	8077.61	Cys106–Cys119,<br>Cys121
19	73.348	1010.34 (2+)	2018.68			
20	76.830	1005.38 (3+)	3013.14			
21	80.045	1034.07 (18+)<br>1094.76 (17+)	18595.26	1–162	18597.17	Cys106–Cys119,<br>Cys66, Cys121, Cys160?
22	84.438	1225.43 (4+)<br>1633.54 (3+)	4897.68	1–45	4897.63	

Table S3. Structure assignments of the fragments obtained by Glu-C digestion of R.

Peak No.	HPLC Reaction time (min)	Observed MS	Estimated MS	Fragment	Expected MS	Cys or SS
1	10.708	539.89 (2+)	1077.80	66–74	1079.36	Cys66
		1078.81 (1+)				
		575.35 (1+)	574.32	159–162	575.73	Cys160
		646.39 (1+)	645.39	109–114	645.62	
		684.51 (1+)	683.51	46–51	683.79	
2	27.580	549.91 (2+)	1097.77	46–55	1098.21	
		659.49 (6+)	3949.63			
		790.66 (5+)				
		857.57 (1+)	856.57			
3	32.492	449.31 (1+)	448.31			
		573.49 (1+)	572.49			
		790.51 (1+)	789.51			
4	35.244	549.88 (2+)	1097.83			
		1098.90 (1+)				
		659.51 (1+)	658.51			
		857.56 (1+)	856.56			
5	44.011	455.98 (1+)	454.38			
		554.46 (1+)	553.46			
		800.61 (1+)	799.61			
6	46.808	681.49 (2+)	1361.56			
		1363.13 (1+)				
7	49.973	644.47 (2+)	1286.96	56–65	1287.42	
		1287.96 (1+)				
8	51.527	560.47 (1+)	559.47			
		645.01 (2+)	1288.02			
9	52.559	590.39 (1+)	589.39			
		703.50 (1+)	702.50			
		816.66 (1+)	815.66			
		929.75 (1+)	928.75	56–62	929.12	
10	54.080	563.48 (3+)	1686.75	75–89	1686.99	
		1688.24 (1+)				
11	55.704	711.80 (4+)	2843.63			
		949.02 (3+)				
		844.68 (2+)	1687.37	75–89	1686.99	
12	56.518	711.81 (4+)	2843.26			
		949.07 (3+)				
13	58.155	544.03 (3+)	1629.11	115–127	1628.02	Cys119, Cys121
		815.47 (2+)				
		1123.66 (2+)	2245.32			
14	59.502	707.77 (2+)	1413.54			
		1415.09 (1+)				
15	62.837	603.65 (4+)	2410.57	90–108	2411.92	Cys106
		804.52 (3+)				
		1206.34 (2+)				
16	63.946	952.63 (2+)	1903.25			
17	68.180	760.99 (2+)	1519.98			
18	69.854	4669.76 (6+)	4669.76			
		935.10 (5+)				
19	73.097	1010.95 (8+)	8077.93			
		1154.75 (7+)				
20	75.823	1010.96 (2+)	2019.92			
21	79.098	1005.00 (3+)	3013.06			
		1508.05 (2+)				
22	81.959	1177.89 (2+)	2353.78			
23	83.061	1233.50 (2+)	2465.00			
24	84.970	1225.45 (4+)	4897.65	1–45	4897.63	
		1633.55 (3+)				
25	86.968	1179.82 (3+)	3536.77			
		1769.54 (2+)				

Table S4. Structure assignments of the fragments obtained by Glu-C digestion of N.

Peak No.	HPLC Reaction time (min)	Observed MS	Estimated MS	Fragment	Expected MS	Cys or SS
1	10.769	751.60 (2+)	1501.20	66−74=159−162	1500.79	Cys66−Cys160
2	27.714	790.68 (1+)	789.68			
3	30.615	**549.91 (2+)**	**1097.81**	**46−55**	**1098.21**	
		659.49 (1+)	658.49			
		857.72 (1+)	856.72			
4	34.177	573.49 (1+)	572.49			
5	43.766	554.57 (1+)	553.57			
		800.76 (1+)	799.76			
6	46.351	681.50 (2+)	1361.01			
7	49.660	**644.47 (2+)**	**1286.95**	**56−65**	**1287.42**	
8	51.290	639.92 (4+)<br>852.95 (3+)	2555.77			
		560.48 (1+)	559.48			
		737.54 (2+)	1473.08			
9	52.430	**465.42 (2+)**<br>**929.81 (1+)**	**928.82**	**56−62**	**929.12**	
		590.39 (1+)	589.39			
		703.51(1+)	702.51			
		816.67 (1+)	815.67			
10	54.130	**563.48 (3+)**<br>**844.15 (2+)**	**1686.30**	**75−89**	**1686.99**	
		713.69 (2+)	1425.37			
11	55.868	711.85 (4+)<br>949.03 (3+)	2843.75			
12	56.886	698.80 (8+)<br>798.25 (7+)<br>931.20 (6+)	5581.17			
13	58.239	544.04 (3+)<br>813.97 (2+)	1625.94			
		668.74 (2+)	1335.48			
		1123.69 (2+)	2245.38			
14	59.676	707.79 (2+)<br>1415.08 (1+)	1413.59			
15	62.491	932.29 (5+)<br>1165.68 (4+)	4656.46			
16	63.011	603.69 (4+)<br>804.63 (3+)	2410.83			
17	64.146	952.63 (2+)	1903.25			
18	69.316	903.75 (12+)<br>985.40 (11+)	10830.73			
19	72.657	**1010.98 (8+)**<br>**1154.73 (7+)**	**8076.10**	**90−158**	**8077.61**	**Cys106−Cys119, Cys121**
20	75.278	1010.97 (2+)	2019.93			
21	78.895	1005.41 (3+)	3013.22			
22	83.573	**1153.57 (16+)**<br>**1230.46 (15+)**	**18441.08**	**1−162**	**18443.17**	**Cys66−Cys160,**<br>**Cys106−Cys119, Cys121**
23	84.487	**1225.48 (4+)**<br>**1633.98 (3+)**	**4897.94**	**1−45**	**4897.63**	
24	86.840	**1179.83 (3+)**<br>**1769.51 (2+)**	3536.50			
